# “I would not go to him”: Focus groups exploring community responses to a public health campaign aimed at reducing unnecessary diagnostic imaging of low back pain

**DOI:** 10.1111/hex.13211

**Published:** 2021-02-18

**Authors:** Sweekriti Sharma, Adrian C. Traeger, Elise Tcharkhedian, Janet Harrison, Jolyn K. Hersch, Kristen Pickles, Ian A. Harris, Chris G. Maher

**Affiliations:** ^1^ Faculty of Medicine and Health Institute for Musculoskeletal Health Sydney School of Public Health The University of Sydney and Sydney Local Health District Sydney NSW Australia; ^2^ Wiser Healthcare Sydney School of Public Health The University of Sydney Sydney NSW Australia; ^3^ Physiotherapy Department Liverpool Hospital Sydney NSW Australia; ^4^ Clinical Governance Department Liverpool Hospital Sydney NSW Australia; ^5^ Faculty of Medicine and Health Sydney School of Public Health The University of Sydney Sydney NSW Australia; ^6^ Ingham Institute for Applied Medical Research South Western Sydney Clinical School UNSW Sydney Sydney NSW Australia

**Keywords:** diagnostic imaging, general public, low back pain, overdiagnosis, public health campaign

## Abstract

**Background:**

Community awareness of the harms of overdiagnosis remains low.

**Objective:**

To evaluate community responses to a public health campaign designed for health service waiting rooms that focuses on the harms of unnecessary diagnostic imaging for low back pain.

**Methods:**

We conducted two focus groups of 19 community members with or without low back pain in Sydney, Australia. This study formed the fourth and final stage of the development process of a public health campaign: (a) initial design, (b) expert review and revision, (c) online experiment and (d) community views & revision. We evaluated reactions to components of the campaign that included digital posters and an information leaflet using strong imagery and messaging about the risk of overdiagnosis. We conducted a qualitative thematic analysis to identify main themes.

**Results:**

Community members reacted with surprise, initial mistrust, and occasionally anger towards imagery and messaging that suggested diagnostic imaging tests could be unnecessary and harmful. With further reflection and discussion, and after reading longer format information about overdiagnosis, the participants found some of the messages informative and useful. Participants appeared to gain a better understanding of the concept of overdiagnosis and the importance of not rushing to imaging.

**Conclusions:**

Public health campaigns including posters and leaflets displayed in waiting rooms could raise awareness about overuse of diagnostic imaging and the harms of overdiagnosis more broadly. However, negative reactions are possible and must be managed carefully.

**Patient or Public Contribution:**

We involved a community participation manager who provided advice on the focus group discussion guide, participant recruitment and manuscript presentation.

## 1 INTRODUCTION

Many patients with low back pain receive diagnostic imaging despite guidelines recommending against it. One systematic review of over 19 million low back pain consultations found that one in four patients in primary care and one in three patients in emergency departments received imaging.^1^ This is problematic because for the majority (90%‐95%) of patients without serious spinal pathology, lumbar imaging will not improve outcomes and can cause unnecessary harms including overdiagnosis and overtreatment.^2^ Trials have found that lumbar imaging findings can increase worry^3^ and more than double the likelihood of having surgery.^4^

There are several reasons why unnecessary diagnostic imaging might happen. These include a clinician's desire to maintain the therapeutic relationship, address a patient's fear or anxiety, and manage their limited consultation time.^5^ Beliefs about imaging appear to be particularly important. Our systematic review of 69 qualitative studies found that clinicians and patients believed imaging could locate the source of pain, legitimize the pain experience and reduce risk of litigation.^6^ Although clinicians were aware of several harms of unnecessary imaging including increased anxiety and overtreatment, patients were not. Interestingly, no qualitative study had explored the beliefs of general community members. While a survey study found community members had misconceptions about imaging for low back pain—more than half expected to have an imaging test during their consultation—^7^ such survey studies do not provide in‐depth understanding of people's beliefs and perspectives. A better understanding of why community members might view imaging as necessary, and how they would react to messaging that discourages the test, could help inform population‐wide approaches to reduce overuse.

One simple, scalable and under‐investigated approach to reducing medical overuse is a public health campaign displayed in health service waiting rooms. A systematic review^8^ of observational and intervention studies found that interventions using waiting room screens (eg TV, tablet, computer) can enhance knowledge about cancer screening, influenza vaccination and contraception in women. However, effects on health behaviour were unclear. Some studies using waiting room screens found increase in healthy behaviours such as uptake of tetanus booster vaccination and polio vaccination, but they used surrogate endpoints.^9^, ^10^ But we still do not know if such interventions reduce medical overuse. To our knowledge, these approaches have not been evaluated in the context of medical overuse.

For the community to engage with public health campaigns, the content should be acceptable and understandable. Nearly 60% of Australian adults have low health literacy, meaning they may have difficulty with understanding and applying the information required for healthcare decision‐making.^11^, ^12^ A Cochrane review suggested that interventions with consumer input in the development phase had better uptake and engagement than those without.^13^ The understandability of public health campaigns could also influence their effectiveness. Consumers can find health promotion messages difficult to understand.^14^ In one study, two‐thirds of patients with cancer had difficulty understanding the information provided to them, leading many of them to seek out alternate information sources that they found more accessible.^15^

We aimed to evaluate community responses to a public health campaign designed for health service waiting rooms. The campaign combines strong imagery on large LCD screens in the waiting area, with messages such as ‘back scans can't heal, they can harm’, and written information about ‐ harms of diagnostic imaging. The campaign materials were developed by a creative innovation agency and refined by researchers in the Wiser Healthcare Research Collaboration,^16^ and aimed to change behaviour by raising awareness of the harms of imaging. This focus group study is part of a larger body of work that has included pilot testing, qualitative studies with patients with low back pain,^17^ Emergency Department doctors,^18^ and GPs,^17^ and a randomized trial in community members.^19^ Our goal was to develop a public health campaign that was acceptable and understandable to community members and that had potential to reduce overuse of diagnostic imaging for low back pain.

## 2 METHODS

Development of the public health campaign has been a multi‐stage process. Campaign messages were determined by back pain researchers (AT, CM, IH) who pitched the idea of increasing public awareness about harms of unnecessary imaging for low back pain to a creative innovation agency. The agency then produced draft versions of the materials—5 posters and a health promotion leaflet. The overarching model of behaviour change was based on behavioural economics, that is, considering the effects of cognitive biases on decision‐making processes. The behavioural cues used in the campaign materials included: framing, loss aversion, anchoring, chunking, status quo bias and suggested alternatives (Box 1).

Box 1 **Behavioural cues included in a health promotion leaflet to reduce unnecessary diagnostic imaging of low back pain**

Framing

‐ Reacting to the same choice differently depending on how it is framed, for example as a loss or a gain^38^
‐ Example from the leaflet: Harms of imaging are listed, but not benefits

Loss aversion

‐ Framing choices to emphasize losses, for example what harms will I avoid if I do not have a scan?^38^
‐ Example from the leaflet: Emphasis is on quantifying the harms of imaging ‘*68 will get a false alarm, 11 will recover more slowly, 1 will have unnecessary surgery, 0 will be better off.’*

Anchoring

‐ Relying heavily on first piece of information presented^38^
‐ Example from the leaflet: Key pieces of information are listed early in the leaflet ‘*Not everyone with back pain needs a back scan. Back scans include x‐ray, CT and MRI. 99% of people who see a GP for low back pain do not need a scan’*.

‘Chunking’

‐ Takes advantage of tendency to remember things that are clustered ^38^
‐ Example from the leaflet: Clinical signs that patients may require a scan and signs that they do not require a scan are grouped together.

Status quo

‐ Easy to do things like they have always been done^38^
‐ Example from the leaflet: Positioned imaging as the **exception not the rule** ‘*Not everyone needs a scan’*.

Suggested alternatives

‐ Suggesting things to do instead of imaging^38^
‐ Example from the leaflet: Directly suggest alternatives ‘*don't rest for too long’*.

**Figure 1:**
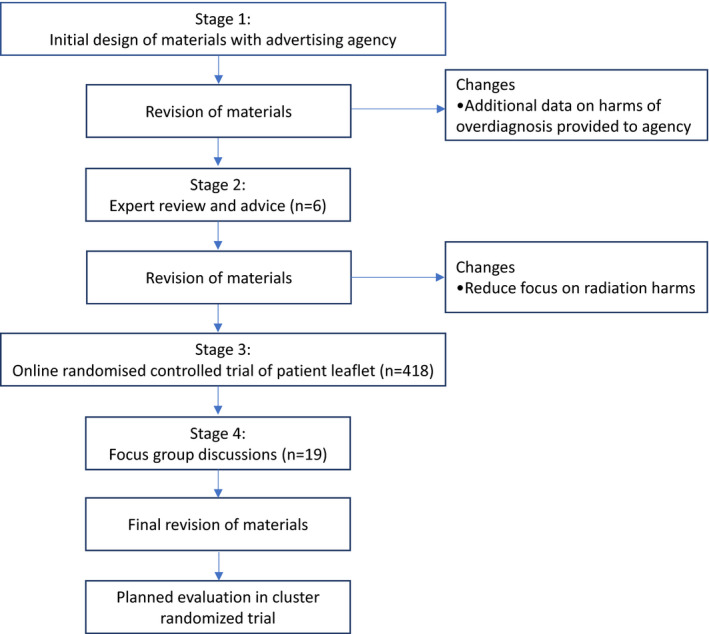
Flow chart for the development of a public health campaign to reduce unnecessary diagnostic imaging of low back pain

Draft versions of the campaign materials were revised based on feedback from experts in back pain care including clinicians and researchers. We tested the revised version of the leaflet in an online randomized controlled trial that found the leaflet changed community members intention to request an imaging test.^19^ The current study was a final step in the larger project, to ensure that these materials are acceptable to members of the community. More details on the steps in the larger campaign project are provided in Table 1 and Figure 1.

**Table 1:** Overview of the public health campaign development and evaluation

Stage 1	This stage involved the design of a draft health promotion strategy in collaboration with a creative innovation agency. Researchers pitched the idea of raising awareness of the harms of unnecessary imaging for low back pain. A key goal of the strategy was to encourage community members to ask questions of their doctor and engage in the decision‐making process. The agency produced draft versions of 5 digital posters and one patient leaflet.
Stage 2	This stage involved revision of the health promotion strategy materials based on expert feedback. The experts included an orthopaedic surgeon, emergency physicians, physiotherapists and back pain researchers. During Stage 2 experts decided that the agency's initial preference to focus on the harms of radiation to deter patients from imaging could not be supported well enough by data (Appendix S1). Following this feedback, the agency shifted focus to the harms of overdiagnosis, including incidental findings that cause worry and increase the risk of unnecessary surgery. Revised versions of the 5 digital posters and patient leaflet were provided to researchers for further testing.
Stage 3	It involved conducting an online randomized trial of the patient leaflet with a sample of 418 community members in Australia.^19^ Stage 3 testing revealed that the leaflet with messages about overdiagnosis could reduce intention to request imaging for low back pain compared with a control leaflet with neutral information about imaging. The patient leaflet also reduced the belief that everyone with low back pain should have imaging.
Stage 4	This stage is the focus of this study and was a qualitative study with 19 community members which aimed to gather their views of Stage 3 versions of the intervention materials which included five posters and a leaflet (Appendix S2).
Stage 5	This stage will be rigorous evaluation in large‐scale randomized trial in a real‐life clinical practice setting.

### 2.1 Design

We conducted a qualitative study using two focus groups of community members. We chose the focus group format to allow opportunities for community members to ask questions and clarify sources of confusion. We wanted to provide the opportunity for participants to hear each other's views and exchange ideas. Another advantage of focus groups is that they shift control of conversation topics from researchers to participants and allow participants to express views, in their own words, on topics not previously anticipated by the researcher.

### 2.2 Ethics

All study procedures were approved by the South Western Sydney Local Health District Human Research Ethics Committee. Ref: 2019/ETH00281. We have reported our methods according to the COREQ statement.^20^

### 2.3 Participant selection and recruitment

We recruited a sample of community members who were over the age of 18 to participate. Community members with or without low back pain were eligible to participate. Those who were not fluent in English were excluded. We aimed for a minimum 2, 8‐person focus groups, based on research suggesting that the majority (two‐thirds) of themes in focus groups studies are often generated from the first group, and more than 80% of the themes can be generated after 2‐3 focus groups.^21^

We conducted the focus groups in a socioeconomically and culturally diverse region of Sydney, Australia. Recruitment of study participants was via the Liverpool Community Representative Network. This Network comprises a Committee of consumer representatives and community members residing in the Liverpool Local Government Area in southwestern Sydney. Network Committee members have a range of consumer advocacy experience and engage in activities to help improve health services in the Liverpool area.^22^ The Network also retains a list of community members who are interested in participating in research. The Community Participation Manager (JH) at Liverpool Hospital advertised the focus groups via the Network using a flyer located in the hospital and by email invitations. Those who were interested contacted JH by phone or email.

The population selected via the Network is representative of the intended audience of the campaign, that is, community members with or without low back pain, from a diverse area of Sydney.

### 2.4 Focus groups and data collection

We conducted focus groups on June 24 (n = 9) and June 28 (n = 10) 2019 at Liverpool Hospital. The focus groups were audio recorded and a researcher (SS) and a research assistant took field notes. Each 90‐minute focus group session was divided into three parts. Part 1 explored participants’ understanding of diagnostic imaging for low back pain. Parts 2 and 3 evaluated reactions to components of the public health campaign (5 digital posters; then one leaflet in hard copy and displayed as a Powerpoint slideshow). Participants were shown the posters for discussion (part 2) before reading the health promotion leaflet (part 3). The groups were facilitated by one of the authors (AT, a researcher with physiotherapy qualifications). Basic demographic information was collected with participants with their consent.

We assessed participants’ health literacy using a single item literacy screener: ‘*How often do you need to have someone help you when you read instructions, pamphlets, or other written material from your doctor or pharmacy? (never, rarely, sometimes, often, always)’*. This literacy screener has been found to perform moderately well in identifying adults with limited reading ability (sensitivity = 54% (95% CI = 47% to 61%) and specificity = 83% (95% CI = 81% to 86)).^23^, ^24^

### 2.5 Components of the public health campaign

#### 2.5.1 ‘Scan your options’ posters

Five different digital posters about overdiagnosis and potential harms of unnecessary imaging (Appendix S2) were shown to participants. Each poster incorporated the slogan ‘Scan your options, not your back’. The posters were designed to be displayed on a 55‐inch digital screen in the waiting room of a hospital emergency department. The prominent messages and images on the posters were as follows:

- Poster 1: ‘Back scans can lead to dangerous and unnecessary treatments’. Image: Surgical scissors.
- Poster 2: ‘Over 2/3 of people who have a back scan will get a false alarm’. Image: X‐ray film of a spine.
- Poster 3: ‘Back scans can't heal‐ they can harm’. Image: X‐ray film of a spine.
- Poster 4: ‘Most people won't benefit from having a scan. It won't find the cause of the pain, and leads to harmful, ineffective treatment’. Image: Photograph of Professor Ian Harris, Orthopaedic Surgeon.
- Poster 5: ‘Ask your doctor: 1. Do I really need a scan? 2. What are the risks? 3. What happens if I don't have a scan?’ Image: X‐ray film of a spine.

#### 2.5.2 Health promotion leaflet

The 2‐page health promotion leaflet had 6 panels of information (Appendix S2). Each page contained cues to emphasize the potential harms of unnecessary imaging for low back pain. The leaflet had a reading Grade Level of 7, that is*,* fairly easy to read; suitable for ages 11‐13 years and above. We pilot tested the leaflet with eight consumers and clinicians before recruiting participants. Consumers and clinicians gave feedback on overall aspects of the leaflet such as the utility, readability and content. We made changes to the leaflet based on their feedback. We tested this revised version of the leaflet in a randomized controlled trial in 418 members of the public and made further changes to wording to improve understandability.^18^

### 2.6 Coding and analysis of qualitative data

We conducted thematic analysis of the transcripts to identify main themes. Four members (SS, AT, KP, JKH) of the research team independently documented salient observations from the transcripts and field notes. The initial impressions of the data, combined with the discussion guide (Appendix S3), formed the basis of the coding framework. The authors (SS and AT) met to discuss the findings and arrive at a final set of themes, which were then reviewed by all authors.

## 3 RESULTS

Of the 19 participants, 12 were female, 14 were born in Australia, 4 had university education, 15 had experienced low back pain in the past 12 months, 11 had undergone an imaging test for back pain in the past (Table 2). 17 of 19 did not need help with written health information, indicating relatively high health literacy. Each group included one member of the Liverpool Hospital Community Participation Network Committee, with experience in consumer advocacy and support. The remainder was members of the public who had expressed interest in participating in research via the Network or in response to the hospital flyer.

**Table 2:** Characteristics of participants

	Participants (n = 19)
Age	
20‐39	2
40‐59	6
60‐79	9
Missing data	2
Sex	
Female	12
Male	7
Born in Australia	
Yes	14
No	5
University education	
Yes	4
No	15
Had low back pain in past 12 months	
Yes	15
No	4
Had an imaging test for back pain (ever)	
Yes	11
No	8

Characteristics of participants in terms of age, gender, country of birth and education were similar in both groups. Participants in Focus Group 1 were more vocal about their opinions and expressed being confronted by the way campaign materials were framed. The tone of the discussion was substantially more negative in Focus Group 1 compared with Focus Group 2. Focus group 2 appeared to be more open to the campaign messaging and spent more time discussing the potential of the campaign to help start conversations between patients and healthcare professionals.

Below we present our key findings with supporting quotes.

### 3.1 Understanding of benefits and harms of diagnostic imaging for low back pain

Participants expressed views that imaging was a valuable tool to help identify the source of low back pain, determine the health of the spine and rule out serious pathology:

‘It gives you answers, whether you like it or not, it gives you answers. So if you’ve had the full scan then you’ve got an answer…I mean some people bung it on as well but [imaging] definitely gives you answers. If you’ve got an answer you know where you stand….Whether your condition is good or bad’ [Male, FG 1]

When asked at the start of the session about potential harms from unnecessary imaging, participants struggled to think of downsides to having the test. Radiation exposure was the most common harm mentioned. The risk that imaging would not show any abnormality that would validate the pain experience was also mentioned:

‘The other thing is that those tests might not show anything, they might not show up any abnormality, so it doesn’t explain the pain at all’ [Female, FG 1]

### 3.2 Response to public health campaign materials

#### 3.2.1 Poster messages were surprising and alarming

##### Messages conflict with beliefs about the value of imaging

In both groups, first impressions of the digital posters were mostly negative. Some participants reacted angrily to the concept of a health service wanting to reduce imaging. They felt the messages were provocative and exaggerated:

‘If I walked into a hospital like that I’d turn around and walk straight back out again, sorry. It’s like, if this is what these people actually believe then I’m out of here because that’s ridiculous, they’re just scaring people to get rid of people’ [Female, FG 1]

The messages on the posters appeared to be in direct opposition to the beliefs expressed by participants in the first part of the focus group. Participants described imaging as an important tool to locate the source of low back pain and associated the test with minimal harm. When they were shown posters suggesting the opposite, the response was defensive:

‘I broke my back! What, so they just don’t do a scan, don’t know about my back and it eventually kills me because my spine eventually collapses? Get real’ [Female, FG 1]

The exception was Poster 5 which suggested three questions to ask a doctor. Participants felt this message was reasonable and aligned with their beliefs about best care of back pain:

‘[the questions to ask your doctor] that’s all you need, nothing else’ [Male, FG 1]

##### Concern about discouraging necessary imaging

There was some concern expressed about the potential for the posters to discourage people from necessary imaging. For example, participants worried that patients might decline imaging after an accident or trauma. Some interpreted the messages to mean that imaging is unnecessary *for all*, rather than the intended message, which was that imaging is unnecessary for those without signs of a serious condition:

‘People like me, I would ignore it (the posters). But it’s the general people. It’s like, as you say, they look at it and they get scared …. They’re the ones that probably need it [imaging]’ [Female, FG 1]

When this detail was later explained in the leaflet, participants in both groups were more comfortable with the concept of discouraging unnecessary imaging.

##### Mixed feelings about using fear to promote health

Some participants felt the posters were designed to generate fear among the public. They likened the approach to public health campaigns for smoking and AIDS that (perhaps more justifiably) relied heavily on the use of fear to promote behaviour change:

*‘*To me [the poster] screams, like, remember [when] AIDS first came out and the grim reaper [advertising campaign]? Scare tactics’ [Female, FG 2‐ all posters]

‘You know what it reminds me of? “Smoking Kills”’ [Female, FG 1]

‘At least it’s true – smoking kills – and there’s been so much research’ [Female, FG 1‐ poster 3]

### 3.3 Scepticism and mistrust of information

#### 3.3.1 Participants’ impressions

##### Unclear if messages are true

Participants expressed doubts about the veracity of data presented in the campaign materials. They were concerned that the figures may not be backed up by research:

‘… where is the statistic coming from?’ [Female, FG 2‐ poster 2]

##### Mistrust of the source of the information

Participants looked at the campaign with scepticism. They expressed disbelief at the messages, including Poster 4 which included a quote from a prominent Orthopaedic Surgeon. To them, the message was interpreted as incongruous and seemed to directly undermine the surgeon's authority:

‘I think that’s false, in fact putting a doctor there, supposedly a doctor and saying that. I would not go to him. That’s one doctor I wouldn’t go to because basically he’s ….. saying most people won’t benefit from having a scan’ [Male, FG1‐ poster 4]

‘This is what I can’t understand. Why would an Orthopaedic Surgeon put himself on a poster with that message?’ [Female, FG 2‐ poster 4]

##### Scepticism about the intent of the intervention

Participants questioned the intent of those involved in producing the campaign materials and the purpose of the campaign itself. Others expressed views that the campaign could be a government initiative to cut hospital expenses:

‘It says we’re spending too much money, that’s what it says’. [Female, FG 1‐ poster 1]

#### 3.3.2 Why participants may have expressed mistrust and scepticism

##### Trusting clinicians over public health messages

Participants discussed having greater trust in their doctor and said they would seek advice from them before believing the messages on the posters:

‘I would look at these posters walking in the hospital and think oh god, roll my eyes, have a chuckle at it and ignore them. I would rely on my GP, I would go to my trusted GP and my experience is mainly with my mum having as many scans as she did have done. What a joke! I’d take a picture of it and probably send it to her and say, look what they’re doing now in the hospital, but I’d ignore it and I’d go straight to my GP’ [Female, FG 1]

##### People have a right to health care

Participants discussed the opinion that this intervention went against the philosophy of healthcare for all:

‘The bottom line is: I will not accept that in future, because the hospital is there for a service and this I find extremely offensive. They should say: we’re here for a service, how can we help you? and then it’s a matter of walking them through the options that they have available. Not saying this, because it says don’t trust your doctor’ [Male, FG 1‐ poster 1]

#### 3.3.3 Some messages were useful and informative

##### Messages raise awareness of why imaging may not be necessary

Participants appeared to understand the reasons why imaging is not recommended and gain a better understanding of the concept of overdiagnosis:

‘Well it’s an awakening, it’s something to [be aware of]. I don’t see the actual threat there, I see … being on notice, being notified’. [Male, FG 2‐ poster 1]

##### Encourages people not to rush into a decision

For some participants, the campaign materials encouraged them to think carefully and avoid rash decisions:

‘Like if you get the flu. You get a backache, you don’t want to rush down and get a scan’ [Female, FG 2]

##### Positive about actions to take

There were some positive views expressed about the specific advice on actions to take to avoid unnecessary imaging such as gentle movement and use of heat. Participants valued some of the content of the leaflet such as three questions to ask doctors and messages about strategies to improve back pain at home:

‘[questions to ask your doctor] should be first, that’s all it should be’ [Male, FG 1‐Leaflet, panel 6]

##### The role of patients vs. healthcare professionals

Participants felt that these campaign materials could be helpful in preparing them to ask questions of their doctors. Messages such as ‘Back scans can lead to dangerous and unnecessary treatments’ were concerning enough that some would ask their doctors about risks of imaging. Participants felt the combination of the leaflet and poster messages would give them more confidence to ask questions. Specifically, the ‘Ask your doctor‐ do I need this test?’ message received almost universal praise across both groups—participants supported components of the campaign that empowered patients with information:

‘I think a lot of it gets down to: people won’t ask questions. We tend to think that these people [doctors] are up here and we’re [patients] down here and we haven’t got the ability to ask the right questions, and I think that’s what we have to get across to the public, it is their right to ask questions about treatment’ [Female, FG 2]

However, participants responded with concerns that asking questions of doctors could promote mistrust.

‘But why should we read the research? This is for the doctors to do. What… we’re supposed to go against doctors’ advice here? That’s basically what it’s telling us to do is go against what your doctors have said’ [Female, FG 1‐ Leaflet]

A participant expressed that the messages appeared to shift blame to patients. They felt the responsibility for imaging decisions and informed consent lay with the doctor.

## 4 DISCUSSION

### 4.1 Main findings

This study identified the potential for public health campaigns to raise awareness about harms of medical overuse and overdiagnosis, but also elicit strong negative reactions from community members. We found that the community members seemed to trust what their family doctor says more than what a poster says. Community members reacted with surprise and initial mistrust, but with further reflection found some of the messages informative and useful (Box 2). Pre‐existing beliefs about the benefits of imaging appeared to heighten negative reactions to some posters that discouraged overuse. However, after viewing the intervention in its entirety (including the accompanying information leaflet) participants appeared to understand the concept of overdiagnosis and the importance of not rushing to an imaging decision. They valued the components of the intervention that highlighted actions to take instead of imaging, and questions to ask of their doctors. This suggests a challenging trade‐off in efforts to better communicate about overdiagnosis: between the positive outcomes of avoiding unnecessary care and the potential for unintended consequences such as mistrust.

Box 2 **Community responses to public health campaign materials**

- Posters were alarming
- Concern about discouraging necessary care
- Negative reaction to use of fear
- Mistrust of the veracity, source and intent of the information
- Trust in doctor's advice over public health campaign messages
- Enlightening about reasons to avoid imaging
- Sensible advice to not rush imaging decisions
- Valuable messages on actions to take and imaging alternatives
- Encourages dialogue between patient and health care professionals

### 4.2 Strengths and limitations

This research has several important strengths. We conducted focus groups in a socioeconomically and culturally diverse area in Australia. We developed public health campaign materials in stages, incorporating feedback from a multidisciplinary team of experts and consumers. We developed the materials to be suitable for people with an average reading level (grade 7), and we have separately demonstrated their potential to influence behavioural intentions regarding imaging.^19^

In terms of limitations, some vocal participants may have influenced the prominence of particular views. However, the facilitator ensured all participants had the opportunity to respond to each question or poster. The participant selection process via the Liverpool Community Representative Network may have introduced bias in our sample. Those who volunteer for research may have different views of imaging and public health communication than those in the general population. For example, participants with experience in consumer advocacy tended to be more vocal in the groups and had strong views against a campaign that discouraged any type of medical care. However, our Community Participation Manager advertised the study broadly via their networks and asked brief screening questions over the phone to ensure the groups were diverse in terms of age, gender, consumer advocacy experience, back pain history and other socio‐demographic characteristics. Finally, although we tried to recruit participants from a diverse population, we recruited only participants with relatively high health literacy.

### 4.3 Comparison with previous research

Several previous studies have examined the role of beliefs in the overuse of lumbar imaging. A systematic review of 1747 patients and clinicians found that overuse might happen because many believe imaging helps identify the source of low back pain and rule out serious pathology.^6^ Our findings in community members suggest such pre‐existing beliefs could influence the way people react to any new information about overuse. Participants in the focus groups who told personal stories of benefits from having imaging appeared, perhaps unsurprisingly, to have the strongest negative reactions to suggestions that imaging tests can be unnecessary or harmful. A systematic review found that mass media campaigns were effective in improving the accuracy of low back pain beliefs among health care providers and the general public, but may not reduce diagnostic imaging rates.^25^ This could be explained by the nature of communication strategies used in the mass media campaigns; most simply encouraged self‐management and addressed myths such as ‘*Bed rest is helpful*’ and ‘*X‐rays and newer imaging tests can always find the cause of pain*’ rather than directly confronting the problem of overdiagnosis. The absence of interventions that can provide robust reductions in imaging rates is evidence that a stronger approach may be needed or that the interventions should focus on clinicians rather than the public.

There is an ongoing debate about using appeals to emotions, such as fear, to promote public health. In a recent article, authors argued that using fear to improve health behaviour could be harmful and may not even be effective.^26^ Some participants in our study likened the imagery used on the posters to health warnings on tobacco products and an infamous ‘scare campaign’ run in Australia to raise awareness of HIV/AIDs in 1987. The fact that a campaign run in the 1980s could be recalled with clarity in 2019 says something about the potential power of using appeals to emotion. More recently a large national anti‐tobacco advertisement campaign in the US depicting fear, graphic images and personal testimonials, had a significant impact on smoking‐cessation behaviour.^27^ Of note is that campaigns using fear or disgust to promote behaviour change were also often accompanied by negative reactions from the public.

There are, however, important differences between those campaigns and the current campaign. Rather than using emotion to encourage a ‘positive’ health behaviour such as safe sex or quitting smoking, the current campaign was discouraging people from asking for, or accepting, a medical test from their doctor. Despite the differences in the campaigns, the effects were similar: campaigns using fear can raise awareness of health issues, including the harms of unnecessary medical care.

These campaigns have also shown that measuring the impact of public health initiatives is not without its challenges. The Australian HIV campaign led to a drastic increase in HIV antibody testing and increased awareness about the spread of the virus.^28^ However, there were also unintended impacts. The nature of the HIV campaign messaging—where a TV advertisement shows the ‘Grim Reaper’ bowling down women and children in a bowling alley as a metaphor for mortality from AIDS—sparked substantial pushback. Some argued that the advertisement sensationalized the illness, provided very little information, scared children, and stigmatized a group who were already facing widespread discrimination.^29^ Appeals to fear in public health should consider measuring not just the impact on health but also the potential unintended impacts on public perception and trust.

Our study suggests that a delicate balance in public health, between maintaining community trust and improving health outcomes, could apply to communicating about overdiagnosis. Such communication is challenging because the public can find the concept of overdiagnosis confusing and there is a risk of negatively affecting those who have been diagnosed already.^30^ We know from previous work that community members can have strong views on the value of a medical diagnosis, react defensively to suggestions about reducing testing, and approach the topic of unnecessary care and overdiagnosis with scepticism.^31^, ^32^ Although participants in our study had negative reactions including mistrust, we observed that the strong images and messages held their attention, promoted vigorous discussion and, after viewing the entire intervention, appeared to increase understanding of the reasons to not rush into imaging. It is unclear whether the same effects could be achieved with a lighter or more ‘positive’ approach to raising awareness, for example through the use of humour. Groups such as Choosing Wisely Canada used humour to communicate the problem of overdiagnosis and too much medicine.^33^ However, like many of the other interventions that have targeted imaging for low back pain, the Choosing Wisely initiative has yet to demonstrate robust effects on imaging rates.^34^, ^35^

### 4.4 Implications for future research and practice

This study provides important insights into why and how people react to strong messaging about overdiagnosis of low back pain. In our study, despite each poster displaying university and health department logos, with one including quotes from a renowned orthopaedic surgeon, community members doubted the veracity of the information. This suggests a need to carefully consider how the source of information about overdiagnosis is communicated. Stark messaging focused on potential harms appears to increase the risk of negative reactions. Future research could compare the impact of strong messaging about harms, neutral information and/or positive appeals (eg using humour, focusing on actions to take) on patient outcomes and community trust. Since some people appeared to trust their own doctor more than generic public health messages, educating clinicians about the harms of overdiagnosis and effective ways of communicating about such harms to their patients may be equally important. No trials have addressed wider population beliefs about imaging using simple public health strategies such as waiting room interventions. We have suggested revisions to our original messaging (Appendix S4). We plan to evaluate the revised messaging in a trial to reduce unnecessary imaging.

Overuse of imaging is a complex phenomenon driven by clinician and patient beliefs, fear of litigation, and patient pressure.^5^, ^6^ This campaign directly targeted only part of the patient‐clinician dyad. However, posters can raise awareness among clinicians indirectly,^8^ and the campaign was designed to target clinicians indirectly via discussions with the patient. Future work could explore whether this happens in practice. Behavioural interventions to reduce overuse are likely to need a multi‐faceted approach and may need to more closely target clinicians.^36^ Nudge‐interventions that encourage changes to clinical behaviour without restricting choice, such as default option nudges in the Electronic Medical Record, have potential to reduce overuse.^36^ More restrictive policy‐based interventions such as removing Medicare funding for ineffective tests and treatments also have potential to reduce overuse.^37^

## 5 CONCLUSIONS

This study suggests that public health campaigns using strong messaging and imagery could help raise awareness about the harms of overdiagnosis and overuse of medical tests but could also generate negative reactions among community members. Strong community beliefs in favour of diagnostic imaging, scepticism about overdiagnosis and anger at the concept of reducing testing could all be barriers to an effective campaign to reduce overuse.

## 6 ROLE OF THE FUNDER/SPONSOR

The funder had no role in the design and conduct of the study; collection, management, analysis and interpretation of the data; preparation, review or approval of the manuscript; and decision to submit the manuscript for publication.

## 7 ADDITIONAL CONTRIBUTIONS

None.

## 8 DATA SHARING STATEMENT

All data relevant to the study is included in the article or uploaded as supplementary information.

## CONFLICT OF INTEREST

CG Maher is supported by a Principal Research Fellowship from Australia's National Health and Medical Research Council (APP1103022) as well as a Program grant (APP1113532) and 2 Centre for Research Excellence grants (APP1134856 and APP1171459). He has received research grants from various government and not‐for‐profit agencies. Flexeze provided heat wraps at no cost for the SHaPED trial that he is an investigator on. His expenses have been covered by professional associations hosting conferences he has spoken at. The remaining authors have no conflicts of interest to declare.

## AUTHOR CONTRIBUTIONS

Traeger, Maher and Sharma: *Concept and design*. Sharma, Traeger, Tcharkhedian, Harrison, Hersch, Pickles, Harris and Maher: *Acquisition, analysis or interpretation of data* and c*ritical revision of the manuscript for important intellectual content*. Sharma and Traeger: *Drafting of the manuscript. Maher and Traeger: Obtained funding and Supervision*.

## Supporting information

Appendix S1‐S4
