# Supplementary material for: “I would not go to him”: Focus groups exploring community responses to a public health campaign aimed at reducing unnecessary diagnostic imaging of low back pain
Source: Health Expect. 2021 Feb 18;24(2):648–58. doi: 10.1111/hex.13211 (PMC8077077; doi:10.1111/hex.13211)
Supplement: Supplementary file 1 — Appendix S1‐S4 [file HEX-24-648-s001.docx]

**eAppendix 1**


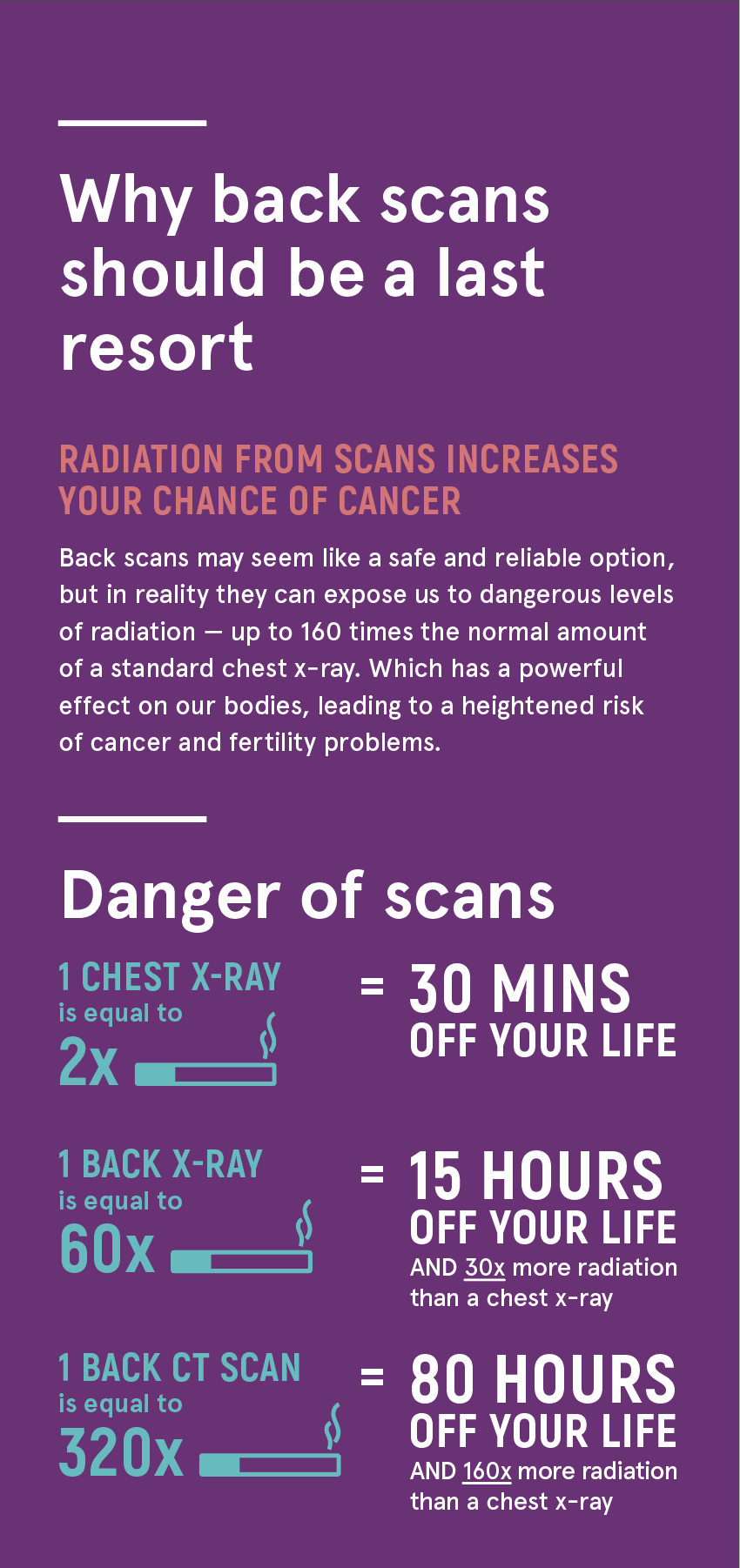


**eAppendix 2**

**Poster 1**

**
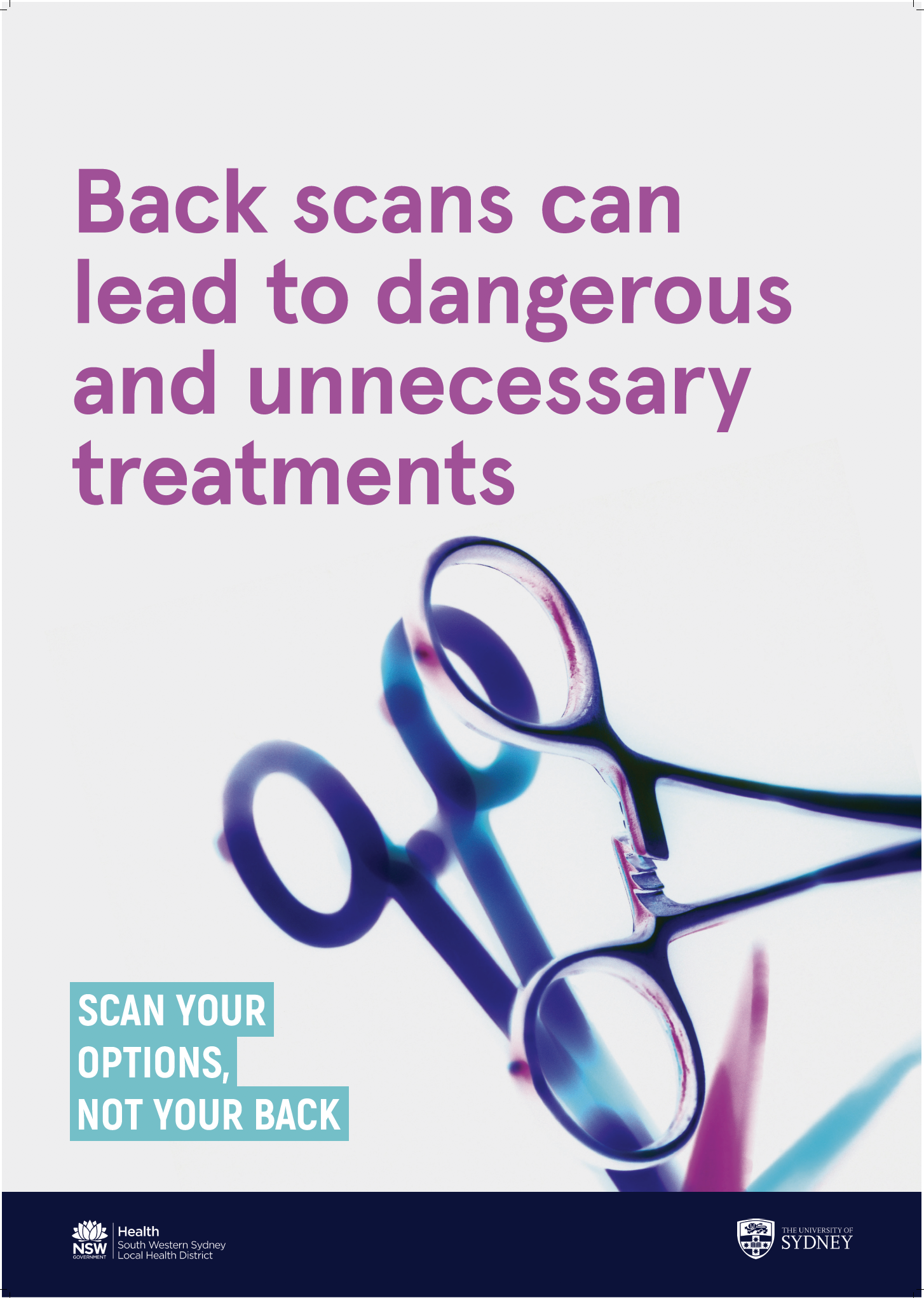
**

**Poster 2**


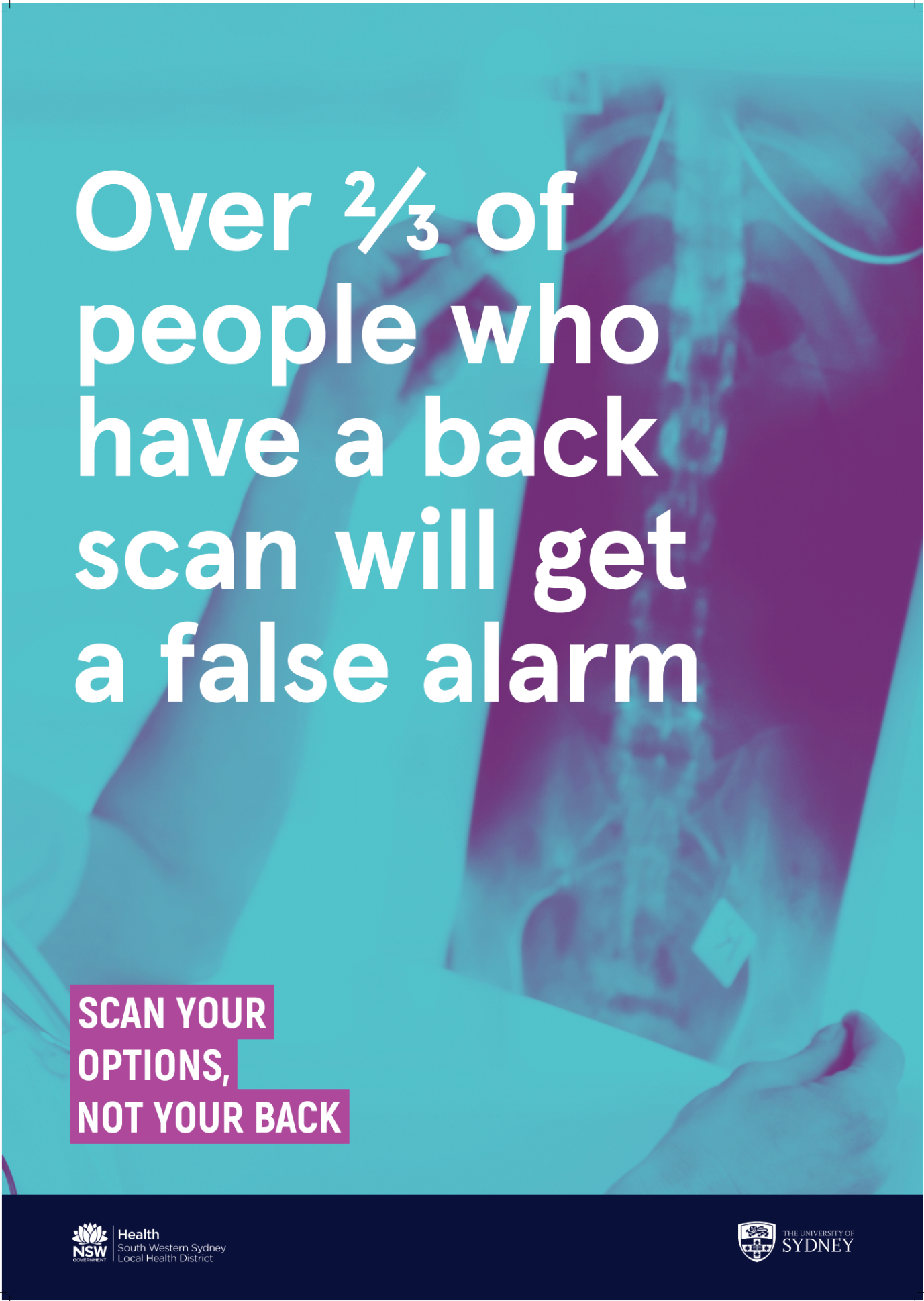


**Poster 3**

**
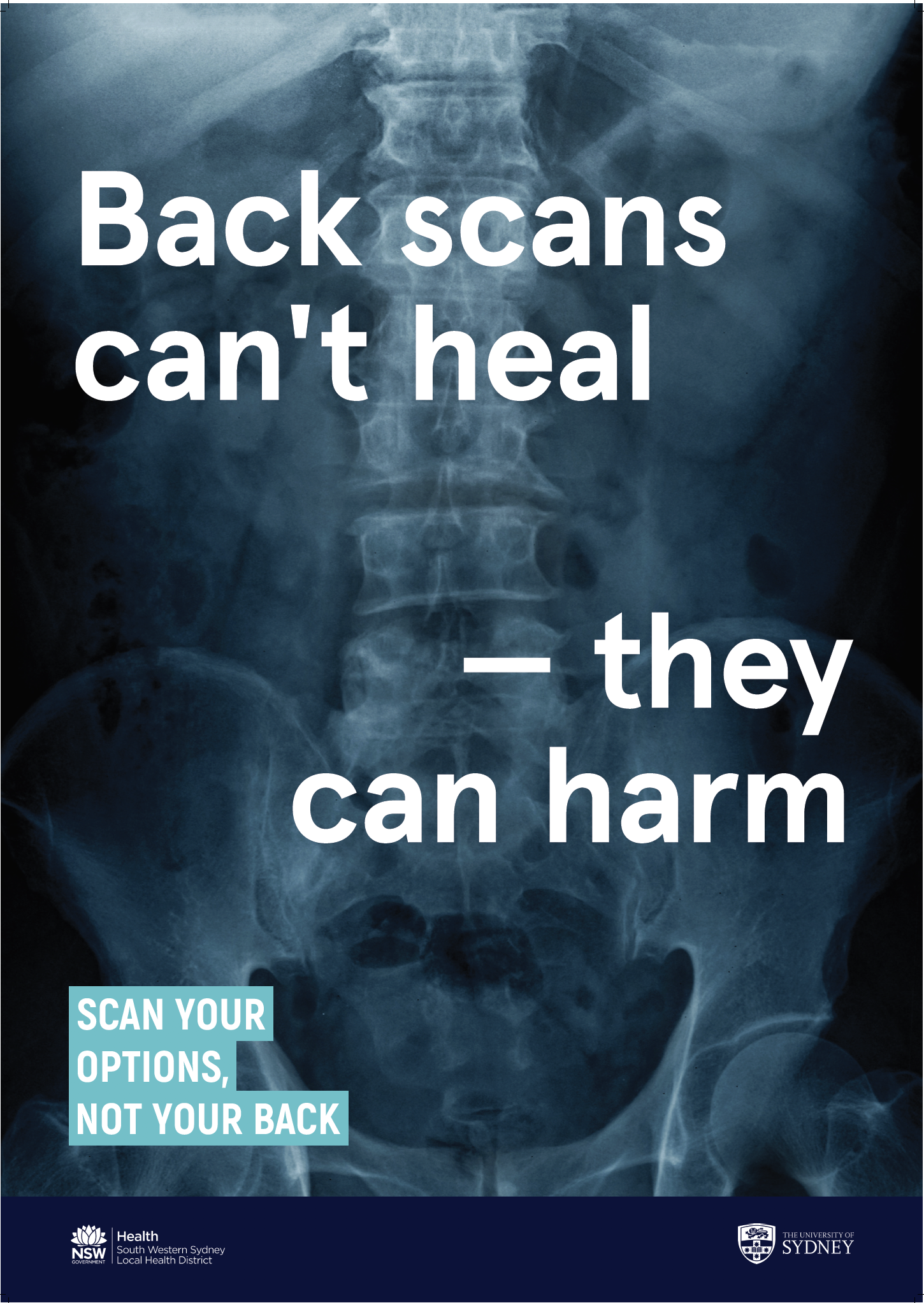
**

**Poster 4**

**
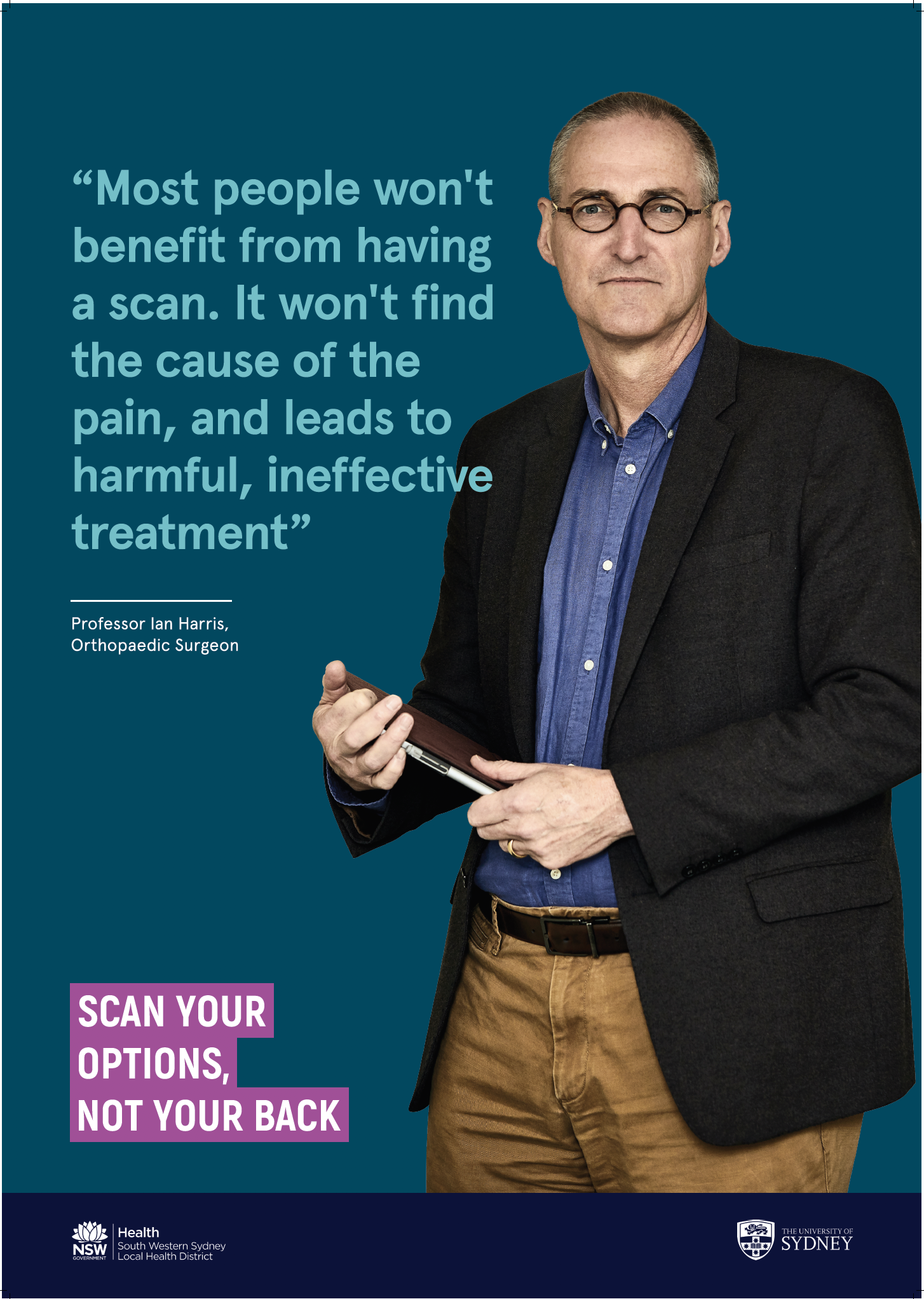
**

**Poster 5**

**
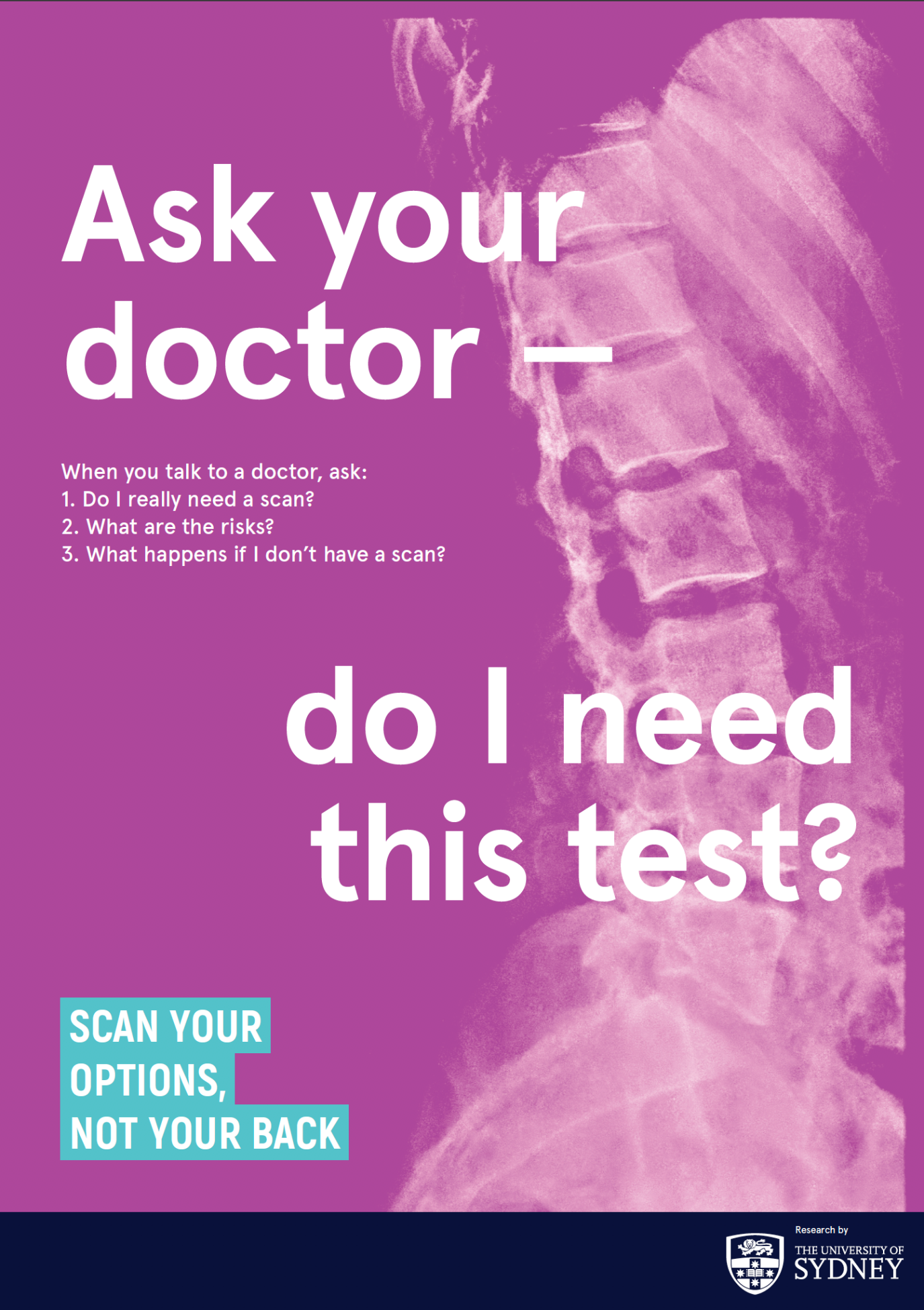
**

**Leaflet**


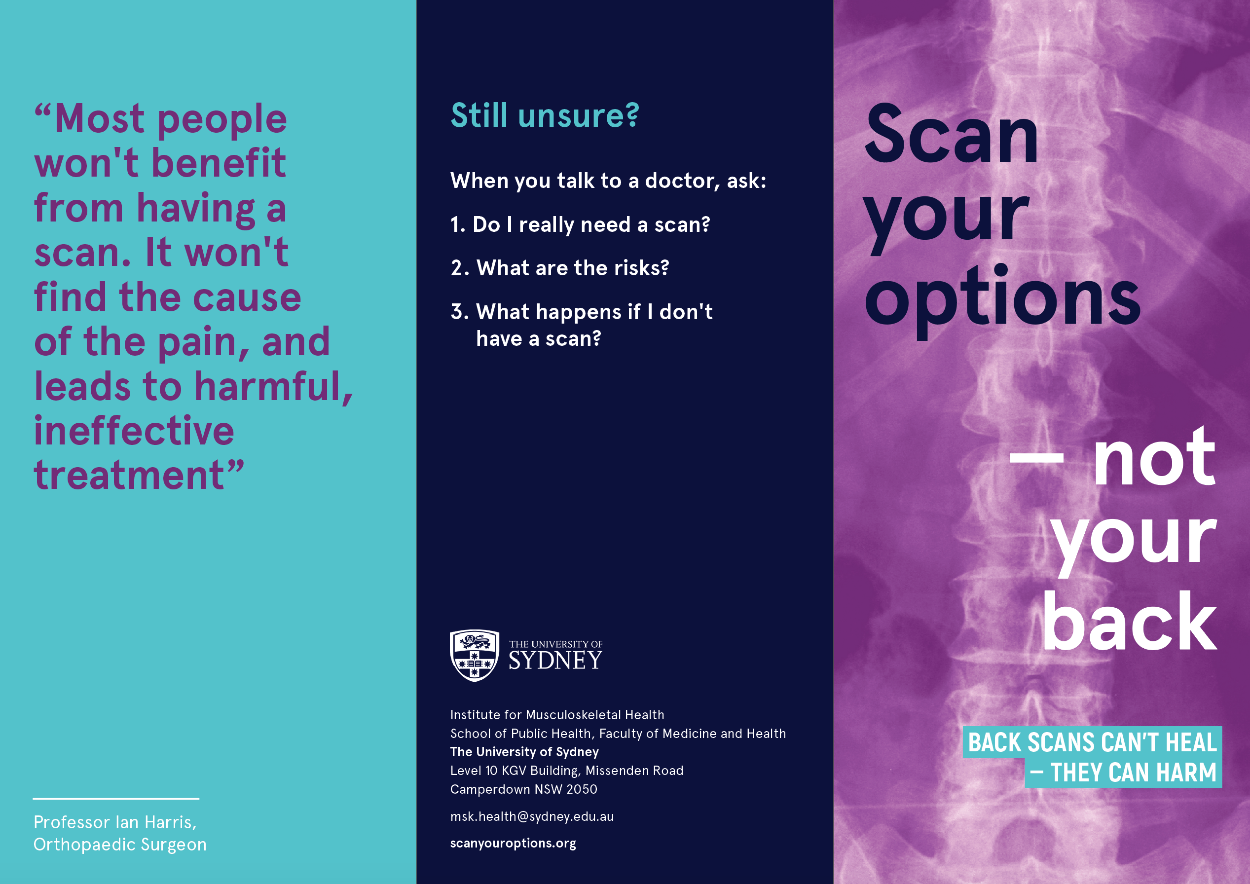


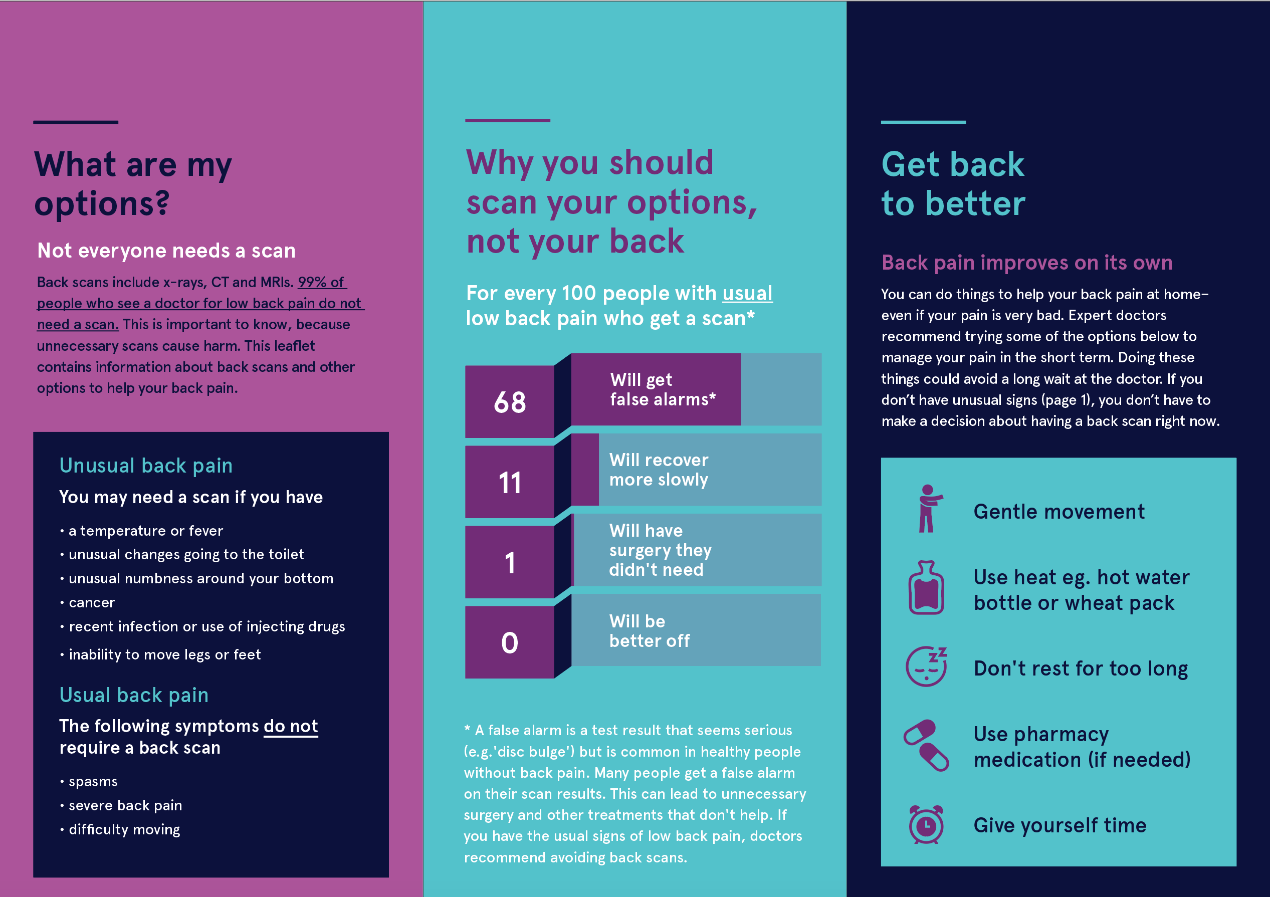


**eAppendix 3**

**COMMUNITY FOCUS GROUP Moderator Guide**

INTRODUCTION [Moderator and observers/note-takers introduce themselves]

- Welcome participants and provide introduction with emphasis on information needed for participants to understand and sign the informed consent form; administer form, answer questions, collect signed forms.
- Explain set-up of session (food, restrooms, timeframe).
- *Explain that staff members cannot give medical advice.*
- We want to find out what you know about health care for low back pain and how you feel about it.
- We will also explain some information that may be new to you. Then we will ask you about your reactions and views regarding this information as well as if and how you think it should be provided to the community.
- The aim of today’s session is to talk about your thoughts and feelings about the information presented and whether you understand this information. There are no right or wrong answers, we are interested in your opinions.

BEFORE STARTING THE SESSION

The recording we make during this session today is for making sure we can accurately summarize our discussion. It will be transcribed into a written record of what is said. No one will hear or see it except for members of the research team.

A few ground rules: We are not looking for a consensus about anything we raise for discussion; we don’t expect you to agree with each other. We would like to hear everyone’s opinions so that we can understand a wide range of opinions. So, it is completely fine to disagree with another person’s opinion but please don’t criticise other people’s opinions.

If you tend to talk a lot, I hope you won’t mind if I sometimes stop you to give someone else a chance to speak. If you are shy, I hope you’ll try to participate. We’d like to hear from everyone. Are there any questions?

Discussion Guide

**9:30am** *INTRODUCTIONS. PARTICIPANTS COMPLETE BASELINE QUESTIONNAIRE SURVEY (WRITTEN)*

**9:40am PART 1: Understanding about diagnostic imaging tests (20min)**

1. What is your understanding of the role of imaging tests (x-Ray, CT, MRI) for somebody with low back pain?
2. How many people do you think need an imaging test for their low back pain? (1%, 10%, 30%, 50%, 99%)
3. What do you see as the advantages of imaging tests? What are the disadvantages?

**10:00am PART 2: Perceptions of an awareness campaign for low back pain (30min)**

| *PRESENTATION OF SCAN YOUR OPTIONS POSTERS* |
| --- |
| *3 slides, one for each poster* |

1. Did you have any thoughts or questions about these public health messages? What about the posters themselves? Was the message clear? Is there any other information you would have liked on a poster?
2. How easy or hard do you think it is for people to understand the reasons for not having an imaging test (x-Ray, CT or MRI)?
3. Do you have any ideas about how best to explain the pros and cons of having an imaging test (x-Ray, CT or MRI) to other people? What could be added, removed or changed from the posters?
4. How does the information on the posters compare with what you already knew?
5. Is there anything else you would like to say on this topic before we finish? Do you have any remaining questions or concerns that we can help you with?

**10:30am PART 3: Perceptions of a decision-making tool for low back pain (30min)**

| *PRESENTATION OF SCAN YOUR OPTIONS LEAFLET*  *1 slide per page of leaflet + hard copies for each participant* |
| --- |
|  |

1. How would you feel if your doctor talked through this brochure with you?
2. Let talk through the brochure, page by page [select different participants read aloud a page]. Do you feel that you understand the information in the brochure? What are your thoughts on what the brochure is saying?
3. We’re now just going to make a list of any advantages you can see about the brochure and also any concerns you may have. So can you see any advantages of the brochure? Do you have any concerns about the brochure?

*FACILITATOR NOTES DOWN ADVANTAGES AND CONCERNS ON A FLIPCHART*

1. Is there any more information you would like to help make a decision about having an imaging test (x-Ray, CT or MRI)?

**eAppendix 4**

**eTable 1: Changes in public health campaigns in response to feedback from the focus groups**

| **Public health campaign** | **Stage 3 version used in focus groups** | **Adapted version** |
| --- | --- | --- |
| Poster 1 | Back scans lead to dangerous and unnecessary treatments | False alarms from back scans can lead to ineffective surgery |
| Poster 2 | Over 2/3 of people who have a back scan will get a false alarm | Many people who have a back scan will get a false alarm |
| Poster 3 | Back scans can’t heal  They can harm | Back scans can cause harm |
| Poster 4 | Most people won’t benefit from having a scan. It won’t find the cause of the pain, and leads to harmful, ineffective treatment. | Back scans rarely find the cause of back pain. Before having the test, ask your doctor what the alternatives are for you. |
| Poster 5 | Ask your doctor  When you talk to a doctor, ask:  1. Do I really need a scan?  2. What are the risks?  3. What happens if I don’t have a scan?  Do I need this test? | Ask your doctor  When you talk to a doctor, ask:  1. Do I really need a scan?  2. What are the risks?  3. What happens if I don’t have a scan?  Do I need this test? |
| Leaflet | Panel 1  Back scans can’t heal   - They can harm   Panel 2  Back scans include x-rays, CT and MRIs. 99% of people who see a doctor for low back pain do not need a scan. This is important to know because unnecessary scans cause harm. This leaflet contains information about back scans and other options to help your back pain.  Unusual back pain  Panel 3  0 will be better off  A false alarm is a test result that seems serious (e.g. ‘disc bulge’) but is common in healthy people without back pain. Many people get a false alarm on their scan results. This can lead to unnecessary surgery and other treatments that don’t help. If you have usual signs of low back pain, doctors recommend avoiding back scans.  Panel 4  You can do things to help your back pain at home even if your pain is very bad. Expert doctors recommend trying some of the options below to manage your pain in the short term. Doing these things could avoid a long wait at the doctor. If you don’t have unusual signs (page 1), you don’t have to make a decision about having a back scan right now.  Give yourself time  Panel 5  “Most people won’t benefit from having a scan. It won’t find the cause of the pain, and leads to harmful, ineffective treatment” | Panel 1  Back scans can cause harm  Panel 2  This is important to know, because having a scan that you did not need can cause harm (see page 2). This leaflet contains information about when you might need a back scan, and when you should try other options first.  Symptoms to look out for  Panel 3  The remainder will not experience any long-term benefit from having the scan.  A false alarm is a scan result that seems serious (e.g. ‘disc bulge’) but is common in healthy people without back pain. Many people get a false alarm on their scan results. This can lead to unnecessary surgery and other treatments that don’t help.  Panel 4  Expert doctors recommend trying some of the options below to manage your pain in the short term. If you don’t have any of the ‘symptoms to look out for’ (page 1), you don’t have to make a decision about having a back scan right now.  Give yourself time. Many recover in 2-4 weeks.  Panel 5  “Back scans rarely find the cause of back pain. Before having the test, ask your doctor what the alternatives are for you.” |
